# Insights into osteoporosis triggered by common antiepileptic drugs via network toxicology, molecular docking and molecular dynamics simulations

**DOI:** 10.3389/fphar.2026.1908344

**Published:** 2026-09-25

**Authors:** Chunpeng Wang, Siyang Gao, Minjie Wu, Bo Liang, Bingbing Wang, Jianye Wang

**Affiliations:** 1 Department of Neurosurgery, The Fifth Affiliated Hospital of Zhengzhou University, Zhengzhou, Henan, China; 2 Department of Reproductive Medicine of Traditional Chinese Medicine, The Third Affiliated Hospital of Zhengzhou University, Zhengzhou, Henan, China

**Keywords:** antiepileptic drugs, epilepsy, molecular docking, molecular dynamics simulation, network toxicology, osteoporosis

## Abstract

**Background:**

Antiepileptic drugs (AEDs), first-line treatments for epilepsy and related seizure disorders, are increasingly recognized as important risk factors for osteoporosis. However, the molecular mechanisms and specific targets underlying AED-induced bone loss remain largely unclear.

**Methods:**

We explored these mechanisms using an integrated strategy combining network toxicology, molecular docking, molecular dynamics simulations, and *in vitro* experiments. Common targets of four AEDs and osteoporosis were identified through database screening, followed by protein-protein interaction network construction, hub gene identification, and functional enrichment analysis. Molecular docking was used to evaluate the predicted binding affinity between AEDs and primary hub targets, and 100-ns molecular dynamics simulations were applied to assess dynamic binding stability. Furthermore, colony formation assays and Western blots were performed in MC3T3-E1 pre-osteoblasts to support the computational predictions.

**Results:**

We identified 47, 50, 46, and 48 potential osteoporosis-related targets for carbamazepine phenobarbital phenytoin (PHT), and valproic acid respectively. These targets were mainly enriched in key signaling pathways and cellular processes associated with bone metabolism. Topological analysis identified four primary hub targets: epidermal growth factor receptor, CASP3, peroxisome proliferator-activated receptor gamma, and PTGS2. AEDs and their targets exhibited favorable binding affinity in the molecular docking simulations (binding energy < −5 kcal/mol). Molecular dynamics simulations revealed heterogeneous dynamic stability among the complexes: PB-CASP3 and PHT-PPARG reached relatively stable conformational equilibrium, whereas CBZ-EGFR displayed notable protein residue flexibility in the equilibrated trajectory, and VPA-PTGS2 sustained persistent structural fluctuations during the simulation period. *In vitro* experiments demonstrated that all AEDs inhibited the clonogenic capacity of MC3T3-E1 cells, and Western blot results further indicated that AEDs differentially modulated the expression and activation levels of their corresponding primary hub targets, which was consistent with the computational predictions.

**Conclusion:**

This study constructs an integrated research framework combining computational prediction and *in vitro* validation to explore potential molecular mechanisms underlying AED-induced osteoblast dysfunction and osteoporosis. The findings highlight candidate targets and provide a preliminary mechanistic basis for future rational clinical medication and skeletal protection in patients receiving long-term AED therapy.

## 1 Introduction

Osteoporosis, a prevalent skeletal disorder characterized by reduced bone mineral density and deteriorated bone microarchitecture, constitutes a major global public health challenge. It markedly increases fracture risk and associated morbidity, and if left untreated, leads to impaired physical function and diminished quality of life (Friedman, 2006; Haseltine et al., 2021). Epidemiological evidence indicates a higher prevalence among postmenopausal women and the elderly, although osteoporosis also affects other populations with diverse risk factors, reflecting its complex and multifactorial etiology (Hsu et al., 2022). Beyond its impact on individual health, osteoporosis imposes a substantial economic burden on healthcare systems due to frequent hospitalizations and increased demand for long-term care (Vandenput et al., 2016). Its pathogenesis involves complex interactions among genetic predisposition, hormonal alterations, and lifestyle determinants (Ahire et al., 2024; Lee and Park, 2018; Marini et al., 2016).

Among various risk factors, long-term medication use has been recognized as an important secondary cause of impaired bone integrity (Aszkiełowicz et al., 2025). Notably, AEDs, which are widely prescribed for epilepsy and other neurological disorders, are increasingly linked to the development of osteoporosis (Elliott, 2009; Wu et al., 2014; Nallaperumal et al., 2025). Accumulating evidence indicates that chronic AED exposure disturbs bone metabolism, lowers bone mineral density, and increases fracture risk (Carbone et al., 2010; Lazzari et al., 2013). These adverse skeletal effects are thought to arise from multiple mechanisms, including induction of cytochrome P450 enzymes, secondary hyperparathyroidism, estrogen deficiency, and imbalance between osteoblast and osteoclast activity (Riva et al., 1996; Goltzman, 2002; Kruse et al., 1980). Although these pathways provide a basic understanding, critical knowledge gaps remain in the comprehensive identification of molecular targets, signaling networks, and their dynamic interactive mechanisms.

Network toxicology is an emerging discipline that predicts chemical toxicity and biological outcomes by using computational simulations and data mining, thereby improving chemical safety assessment and reducing dependence on animal experiments (Xie et al., 2024; Li et al., 2022). With the support of comprehensive biological databases, molecular docking enables high-throughput screening and quantitative assessment of binding affinities in biomolecular systems, facilitating the identification of potential drug targets and interaction patterns (Huang and Zou, 2010; Sim et al., 2025). Similarly, molecular dynamics simulation uses numerical algorithms to solve Newtonian equations of motion, enabling atomic-level characterization of time-dependent conformational changes and quantitative assessment of binding free energy and key residue contributions.

Unlike previous studies that examined individual drug-target pairs in isolation or utilized limited datasets, this study integrates network toxicology, molecular docking, molecular dynamics (MD) simulations, and *in vitro* cell experiments to systematically identify primary hub targets and explore biological processes associated with AED-induced osteoporosis. Additionally, colony formation assays and Western blot analyses in MC3T3-E1 pre-osteoblasts assessed protein-level changes corresponding with the regulatory effects of AEDs on cell proliferation and primary hub target protein expression (Figure 1). These findings provide preliminary insights into the diagnosis and management of AED-related bone loss and may inform future research on optimizing AED regimens and developing adjuvant therapies to maintain skeletal health in patients undergoing long-term antiepileptic treatment. The computationally predicted targets and *in vitro* observations require further investigation in animal models and clinical cohorts to assess their translational potential.

**FIGURE 1:**
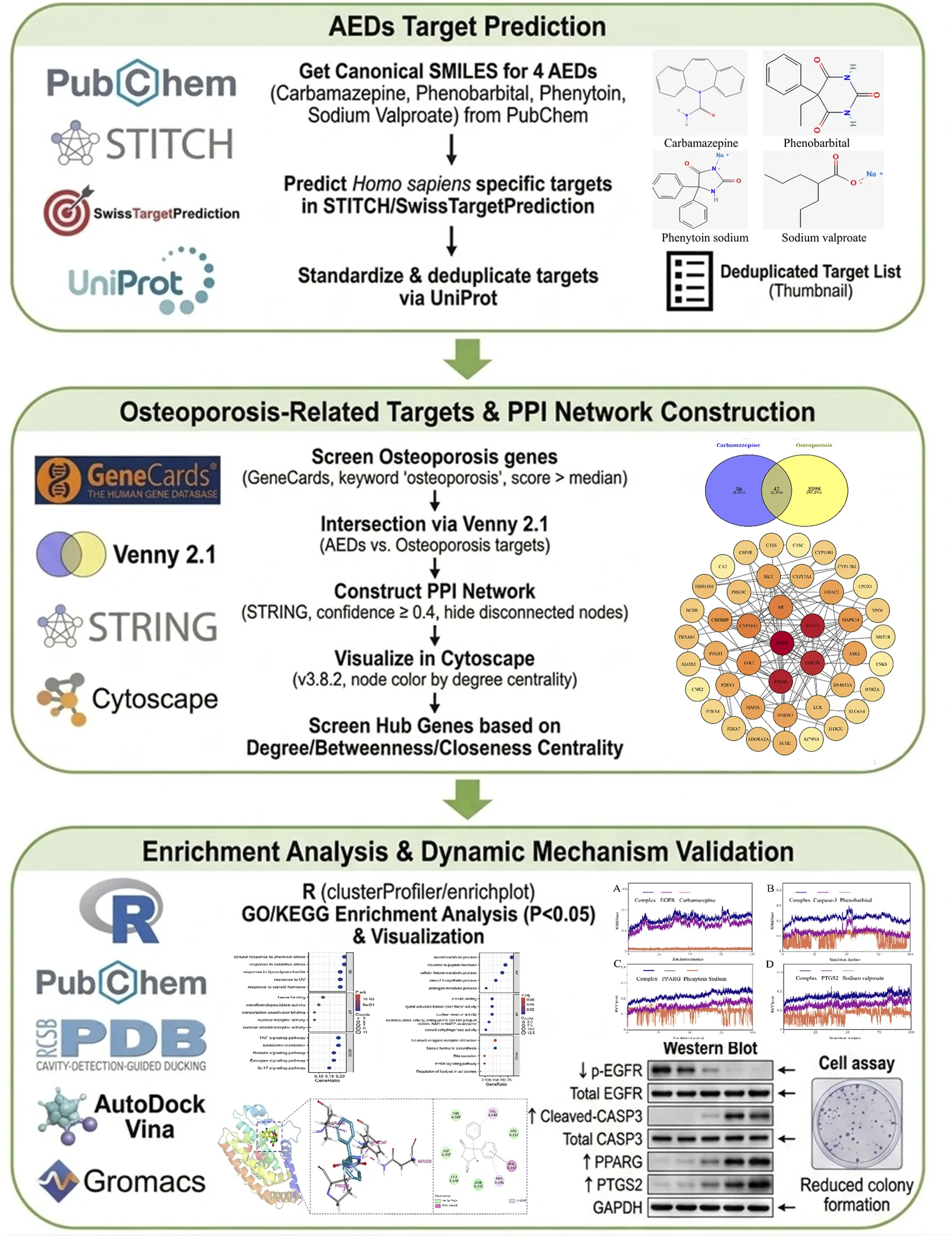
Schematic workflow of the study.

## 2 Materials and methods

### 2.1 Target prediction of AEDs

The chemical structures of four commonly used AEDs (carbamazepine, phenobarbital, phenytoin, and valproic acid) were retrieved from the PubChem database (https://pubchem.ncbi.nlm.nih.gov/) and exported as canonical SMILES strings. All database retrieval and target prediction workflows were performed in May 2026. Potential human protein targets of each AED were predicted via STITCH v5.0 (http://stitch.embl.de/) and SwissTargetPrediction (2019 release, http://swisstargetprediction.ch), with the search species restricted to *Homo sapiens*. In STITCH, only chemical-protein interaction pairs with a combined confidence score ≥0.4 were retained, while lower-scoring pairs were discarded. For SwissTargetPrediction, candidate targets with a prediction probability ≥0.1 were preliminarily filtered, and the top 50 targets sorted by descending prediction probability were retained for downstream analysis to exclude low-relevance predictions. Raw target entries from both databases were imported into the UniProt database (https://www.uniprot.org/, release 2026_02) for standardization of official protein identifiers, and duplicate entries were removed to ensure data consistency. The raw target counts per database, deduplicated statistics, and the complete list of unique drug targets are summarized in Supplementary Table S1.

### 2.2 Screening of osteoporosis-related targets

Osteoporosis-associated human disease genes were retrieved from GeneCards version 5.26 (https://www.genecards.org/) in May 2026 using the keyword “osteoporosis”. The GeneCards relevance score was used to quantify gene-disease relevance. The median relevance score of all retrieved genes was calculated as the screening threshold; only genes scoring above this median were retained as osteoporosis-related candidates and subsequently intersected with the predicted drug targets (Shi et al., 2023; Mu et al., 2025). This data-driven threshold was selected to avoid arbitrary cutoff selection and to ensure balanced sensitivity and specificity in disease-gene inclusion.

### 2.3 Identification of shared targets and PPI network construction

Venny 2.1 (https://bioinfogp.cnb.csic.es/tools/venny/) was utilized to obtain intersecting targets of AED-related and osteoporosis-related genes. These common targets were submitted to the STRING database (https://string-db.org/, Version: 12.0, in May 2026) for PPI construction. The organism was specified as *H. sapiens*, retaining all default interaction sources (text mining, experiments, databases, co-expression, neighborhood, gene fusion, and co-occurrence) and applying the default medium confidence threshold (score ≥0.4) without modification. No additional filters or advanced settings were applied. The resulting network was exported in simple interaction format (SIF), and isolated nodes (proteins with no interactions above the threshold) were excluded during network visualization in Cytoscape 3.8.2. Node color intensity was scaled by degree centrality to reveal core hub proteins.

### 2.4 Identification of hub genes

Hub genes were screened using the CytoNCA plugin in Cytoscape based on three topological parameters: degree, betweenness centrality, and closeness centrality. Degree quantifies the number of direct edges connected to a specific node, reflecting the extent of a protein’s immediate interactions within the network. Betweenness centrality measures the fraction of all shortest paths between node pairs that traverse a given node, thereby indicating its role as a pivotal conduit for information flow. Closeness centrality, the reciprocal of the mean shortest path length from a target node to all others, captures the transmission efficiency inherent to that protein.

After sorting all network nodes by these metrics, genes falling within the top 10% for all three indices were preliminarily designated as hub gene candidates. According to previous network biology research, degree centrality serves as a fundamental metric for evaluating a gene’s relative importance in a network (Gu et al., 2015). A secondary filtering process was subsequently performed based solely on the degree quantile distribution of these candidates, retaining those with the highest degree values as definitive core hub genes. Detailed information on shared candidate targets is summarized in Supplementary Table S2.

### 2.5 GO and KEGG enrichment analysis

Gene Ontology (GO) functional enrichment and Kyoto Encyclopedia of Genes and Genomes (KEGG) pathway enrichment analyses were conducted using the clusterProfiler package (v.4.12.0) in R 4.4.0 (R Core Team, 2024). The org. Hs.e.g.,.db package (v.3.18.0) facilitated gene ID conversion and annotation. GO terms were accessed via the GO. db package (v.3.18.0) in May 2026, while KEGG pathway data were obtained from the KEGG REST API (https://rest.kegg.jp/) during the same month. Terms with adjusted *p* < 0.05 (Benjamini–Hochberg correction) were regarded as statistically significant. Visualization utilized enrichplot (v.1.22.0) and ggplot2 (v.3.5.0), with all data from GO and KEGG enrichment analyses compiled in Supplementary Table S3.

### 2.6 Molecular docking

Two-dimensional structures of AED ligands were retrieved from PubChem and converted to three-dimensional conformations using ChemOffice 20.0 (PerkinElmer, Waltham, MA, United States of America). All 3D structures underwent energy minimization and were exported as MOL2 files. Crystal structures of target proteins were sourced from the RCSB PDB database (https://www.rcsb.org), including EGFR (PDB ID: 1M17), Caspase-3 (PDB ID: 6PDQ), PPARG (PDB ID: 9OLC), and PTGS2 (PDB ID: 5IKR). A standardized preprocessing protocol ensured docking accuracy, with missing terminal residues and surface-exposed loops lacking electron density retained as deposited, due to their distance from the orthosteric binding pockets. For residues exhibiting alternate conformations, the highest-occupancy rotamer was selected. Native ligands, cofactors, and crystallographic waters were removed to prevent steric interference and facilitate the evaluation of direct protein-ligand interactions. Comprehensive details regarding PDB ID selection, biological assembly, resolution, and conformational states for each target are provided in Supplementary Table S4. Original docking source files, including receptor and ligand PDBQT files, docking log files, and 2D/3D interaction diagrams for all four drug-target complexes, are available as Supplementary File S1.

Protein structures were preprocessed using PyMOL to remove all crystallographic water molecules, phosphate ions, and non-protein heteroatoms. Polar hydrogens were added assuming physiological pH (7.4), and Kollman partial charges were assigned to protein receptors using AutoDock Tools (ADFR Suite 1.5.6). All antiepileptic ligands used for target prediction, molecular docking and molecular dynamics simulations were retrieved as neutral free molecules from PubChem; no sodium counterions or ionic dissociated forms were introduced into any computational workflow. Ligands were processed in AutoDockTools to define rotatable bonds and assign Gasteiger partial charges, then converted to the required PDBQT format for AutoDock Vina docking.

To ensure consistent computational treatment across docking and MD workflows, the following unified molecular preparation criteria were established: (1) Protonation: All macromolecules and ligands were prepared at pH 7.4 according to canonical residue protonation rules, with ligands maintained in their native protonation states. (2) Ionization: Sodium cations from phenytoin sodium and sodium valproate were excluded to represent their neutral dissociated form under physiological conditions. (3) Stereochemistry: Intrinsic stereocenters and double-bond geometries of ligands, along with native protein crystal backbones, were fully preserved without chiral inversion or racemization during structural optimization. (4) Charge scheme: Kollman and Gasteiger partial charges were adopted for protein and ligand docking calculations, respectively, while AM1 BCC (ligand) and Amber14 S B (protein) force field charges were employed in downstream MD simulations, with all solvated systems neutralized with 0.15 M NaCl.

Molecular docking was conducted using AutoDock Vina 1.1.2, with the docking grid box centered on the orthosteric binding site of each target. For EGFR (1M17), PPARG (9OLC), and PTGS2 (5IKR), the binding sites were defined based on the positions of co-crystallized ligands (erlotinib, FTX-6746, and mefenamic acid, respectively). In the case of Caspase-3 (6PDQ), which lacks a co-crystallized ligand, the active site was determined based on the catalytic dyad residues Cys163 and His121, with the grid center calculated as the geometric center of these two residues. Random seeds were automatically generated by the Vina engine, and specific values were recorded from the output logs to ensure traceability of the reported poses. The conformation with the lowest docking score was selected for interaction analysis and subsequent MD simulation. Ligand–residue interactions were visualized using PyMOL and Discovery Studio 2019, including two-dimensional interaction diagrams and three-dimensional conformation analysis. Details regarding the grid box center coordinates, dimensions, exhaustiveness, and random seed settings are listed in Supplementary Table S5, along with detailed ligand information (PubChem CID and canonical SMILES) and docking scores.

### 2.7 Molecular dynamics simulation

All 100-ns MD simulations were conducted using GROMACS 2022.2. Protein parameters were assigned utilizing the Amber14 S B force field, while ligand parameters were generated with Antechamber, employing AM1-BCC partial charges and GAFF2 atom types. The resulting ligand parameters were converted into GROMACS-compatible topology files using ACPYPE, ensuring that both protein and ligand parameters derived from a unified AMBER force field framework. Each protein–ligand complex was solvated in a dodecahedral simulation box using the TIP3P water model, maintaining a minimum distance of 1.2 nm between the solute surface and the box boundary. Sodium (Na^+^) and chloride (Cl^−^) ions were introduced to neutralize the system and achieve a physiological salt concentration of 0.15 M NaCl, with ion parameters compatible with the TIP3P water model according to the Joung–Cheatham parameters. Specific box dimensions, initial volumes, water molecule counts, ion numbers, and total atom counts are detailed in Supplementary Table S6.

Periodic boundary conditions were applied, and long-range electrostatic interactions were calculated using the particle mesh Ewald (PME) algorithm. Energy minimization was performed via the steepest-descent algorithm until the maximum force fell below 1,000 kJ mol^–1^·nm^–1^. The systems were then sequentially equilibrated under NVT and NPT ensembles for 200 ps each. During equilibration, position restraints with a force constant of 1,000 kJ mol^–1^·nm^–2^ were applied to the protein and ligand in all three spatial dimensions; these restraints were subsequently released during the 100-ns production runs. The temperature was maintained at 298 K using the Nosé–Hoover thermostat with a coupling constant of τt = 0.2 ps, while the pressure was maintained at 1 bar using the Parrinello–Rahman barostat with a coupling constant of τp = 2.0ps Production simulations were executed for 100 ns under NPT conditions with a 2-fs integration time step, employing the Verlet cut-off scheme. Long-range electrostatic interactions were treated using the PME method, with cut-off distances for both van der Waals and short-range Coulomb interactions set at 1.2 nm. Bonds involving hydrogen atoms were constrained using the LINCS algorithm. Trajectory frames were saved every 10 ps, and each complex was simulated as a single trajectory.

Hydrogen bonds were identified using the default geometric criteria implemented in GROMACS. The protein–ligand distance was calculated as the center-of-mass distance between the protein and the ligand, while the docking-site-ligand distance was defined as the center-of-mass distance between the predicted docking-site residues and the ligand after trajectory alignment. Before distance and RMSD analyses, trajectories were corrected for periodic-boundary artifacts.

For quantitative trajectory analysis, the equilibrated interval from 50 to 100 ns was utilized to summarize structural parameters. Complex RMSD, protein RMSD, ligand RMSD, RMSF, radius of gyration (Rg), solvent-accessible surface area (SASA), protein–ligand distance, docking-site–ligand distance, and hydrogen-bond numbers were calculated and reported as mean ± standard deviation (SD). Protein RMSD calculations involved first least-squares fitting the protein backbone atoms (Cα) to the corresponding backbone atoms of the initial reference structure to eliminate global translational and rotational motions, followed by RMSD computation using the same fitted backbone atoms. For ligand RMSD calculations, the trajectory was aligned to the protein backbone before calculating ligand RMSD to assess ligand displacement relative to the protein environment. MM-PBSA binding free-energy calculations were performed using gmx_MMPBSA with snapshots extracted from the same equilibrated trajectory interval. The total binding free energy was computed as the sum of van der Waals, electrostatic, polar solvation, and nonpolar solvation contributions.

### 2.8 Cell culture and drug treatment

The mouse preosteoblastic cell line MC3T3-E1 was cultured in α-MEM supplemented with 10% fetal bovine serum and 1% penicillin-streptomycin at 37 °C in a 5% CO2 environment. CBZ and PB were dissolved in anhydrous dimethyl sulfoxide (DMSO), while sodium phenytoin and sodium valproate were dissolved in sterile water due to their ionic nature and high aqueous solubility. All stock solutions were subsequently diluted in culture medium. The final DMSO concentration was standardized across all treatment groups and maintained below 0.1% to mitigate solvent cytotoxicity. Working concentrations (CBZ 20/40 μM, PB 20/40 μM, PHT 10/20 μM, VPA 500/800 μM) were selected based on clinically relevant therapeutic concentration ranges reported in previous experimental studies (Hahn et al., 1972; Wang et al., 2023; Feldkamp et al., 2000; Fan et al., 2020), and were kept lower than the upper concentration limits applied in analogous *in vitro* cellular assays. No precipitation or solubility issues were observed throughout the experiments. Information regarding cells and culture conditions is summarized in Supplementary Table S7.

### 2.9 Cell viability assay

Cell viability was assessed using the Cell Counting Kit-8 (CCK-8) assay according to the manufacturer’s protocol. MC3T3-E1 cells were seeded into 96-well plates at a density of 5,000 cells per well in α-MEM supplemented with 10% fetal bovine serum, then incubated overnight (12–16 h) to ensure complete adherence. Following attachment, the cells were treated with serially diluted antiepileptic drugs (AEDs) (CBZ: 20, 40 μM; PB: 20, 40 μM; PHT: 10, 20 μM; VPA: 500, 800 μM) or an equal volume of DMSO vehicle control (final DMSO concentration ≤0.1%) for 24 h and 48 h. After treatment, the original culture medium was replaced with 100 μL of fresh serum-free medium, followed by the addition of 10 μL of CCK-8 reagent to each well. Plates were incubated at 37 °C in a humidified incubator with 5% CO_2_ for 2 h. Absorbance at 450 nm was measured using a microplate reader (Thermo Fisher Scientific, Waltham, MA, United States of America). Cell-free wells containing medium and CCK-8 reagent served as blank controls to eliminate background absorbance. Relative cell viability (%) was calculated using the following formula [(A_450__treatment − A_450__blank)/(A_450__vehicle − A_450__blank)] × 100%. Three technical replicate wells were utilized for each experimental condition, and all treatments were performed across three independent biological replicates.

### 2.10 Colony formation assay

The colony formation assay was conducted to evaluate the proliferative and clonogenic capacity of MC3T3-E1 preosteoblasts. Cells were seeded into 6-well plates at a density of 500 cells per well and incubated for 24 h to allow for complete cell attachment. The culture medium was then replaced with fresh media containing AEDs at the specified concentrations: CBZ (20 μM, 40 μM), PHT (10 μM, 20 μM), PB (20 μM, 40 μM), and VPA (500 μM, 800 μM). An equal volume of DMSO was added to the medium of the vehicle control group, maintaining the final DMSO concentration below 0.1%. Cells were continuously cultured for 14 days, with the culture medium refreshed every 3 days.

After 14 days, the culture medium was discarded, and cell monolayers were gently rinsed twice with pre-cooled phosphate-buffered saline (PBS). Cells were fixed using 4% paraformaldehyde at room temperature for 15 min, followed by staining with a 0.1% (w/v) crystal violet aqueous solution for 30 min. Excess stain was washed away with slow-running tap water, and plates were allowed to air-dry at room temperature. Visible cell colonies containing more than 50 cells were manually counted under a light microscope.

Each experimental group consisted of three replicate wells for each independent experiment, and the entire colony formation assay was repeated three times as independent biological replicates (n = 3). The clonogenic survival rate for each drug-treated group was normalized to the vehicle control group, where the survival rate was defined as 100%. The formula for calculating the clonogenic survival rate is as follows: Clonogenic survival (%) = (Mean colony number of drug group/Mean colony number of vehicle control group) × 100%.

### 2.11 Western blot analysis

To explore the underlying molecular mechanisms, MC3T3-E1 cells were exposed to four AEDs at designated working concentrations (CBZ, 40 μM; PB, 40 μM; PHT, 20 μM; VPA, 800 μM) for 48 h. All experiments included a minimum of three independent biological replicates (n = 3), initiated from separate cell cultures, followed by drug stimulation, cell lysis, total protein extraction, protein quantification, SDS-PAGE separation, membrane electrotransfer, primary and secondary antibody incubation, chemiluminescence signal acquisition, and densitometric analysis. Vehicle control and drug-treated groups were prepared in parallel for each independent experiment.

Total cellular protein was extracted using RIPA lysis buffer supplemented with protease and phosphatase inhibitor cocktails. Protein concentrations were determined via the BCA assay. Equal amounts of protein (20–30 μg per lane) were separated by SDS-PAGE and transferred onto PVDF membranes. Membranes were blocked with 5% non-fat dry milk diluted in TBST for 1 h at room temperature, followed by overnight incubation at 4 °C with primary antibodies targeting EGFR, phospho-EGFR, Caspase-3, cleaved-Caspase-3, PPARG, and PTGS2. Detailed information, including antibody catalogue numbers, clones, host species, and antigen phosphorylation sites, is summarized in Supplementary Table S8; GAPDH was used as the housekeeping loading control.

Target proteins and GAPDH were detected sequentially on identical PVDF membranes from the same biological sample batches. After imaging target bands, membranes were rinsed with TBS for 5 min, stripped with a commercial antibody stripping buffer under gentle agitation at 37 °C for 30 min, and re-probed with anti-GAPDH antibody. All protein lanes for target proteins corresponded perfectly with the respective GAPDH lanes. Following thorough TBST washing, membranes were incubated with HRP-conjugated secondary antibodies (1:5,000 dilution) for 1 h at room temperature. Immunoreactive protein bands were visualized using ECL chemiluminescent substrate and captured with a Bio-Rad chemiluminescence imaging system.

Multiple exposure times were acquired for each membrane. Densitometry analysis was performed on images without pixel saturation, exhibiting low background and falling within the instrument’s linear detection range; the built-in saturation verification tool confirmed no saturated pixels in quantified target and GAPDH bands. Phospho-EGFR levels were normalized to total EGFR, and cleaved Caspase-3 was normalized to total Caspase-3, with GAPDH serving as the global loading control for total protein quantification. All quantitative plots and statistical analyses were generated based on normalized densitometric values.

### 2.12 Statistical analysis

Quantitative data from three independent biological replicates were presented as mean ± SD. The Shapiro–Wilk test assessed the normality of data distributions, while Levene’s test evaluated variance homogeneity for normally distributed datasets. Pairwise comparisons between the vehicle control and treatment groups were performed as follows: a standard independent samples t-test was applied for normally distributed data with equal variances, whereas the independent samples t-test with Welch’s correction was used for normally distributed data with unequal variances; datasets failing the normality test were analyzed using the non-parametric Mann–Whitney U test, regardless of variance homogeneity. Bonferroni correction was applied to adjust *p-*values to reduce type I error during multiple pairwise comparisons within the same experimental group. Differences with *p* < 0.05 were considered statistically significant.

## 3 Results

### 3.1 Effects of carbamazepine on osteoporosis

A total of 47 potential targets were identified that are shared between CBZ and osteoporosis (Figure 2A). GO enrichment analysis indicated that these targets are primarily involved in biological processes such as response to xenobiotic stimulus, peptide hormone signaling, steroid hormone biosynthesis, and growth hormone receptor signaling via the JAK–STAT pathway (Figure 2B). Cellular component analysis localized these targets mainly to the nuclear envelope and integral components of presynaptic membranes, while molecular function analysis revealed enrichment in phosphatase binding. KEGG pathway analysis further demonstrated significant enrichment in the serotonergic synapse, steroid hormone biosynthesis, ovarian steroidogenesis, and calcium signaling pathways, suggesting that these pathways may be linked to CBZ-related alterations in bone metabolism.

**FIGURE 2:**
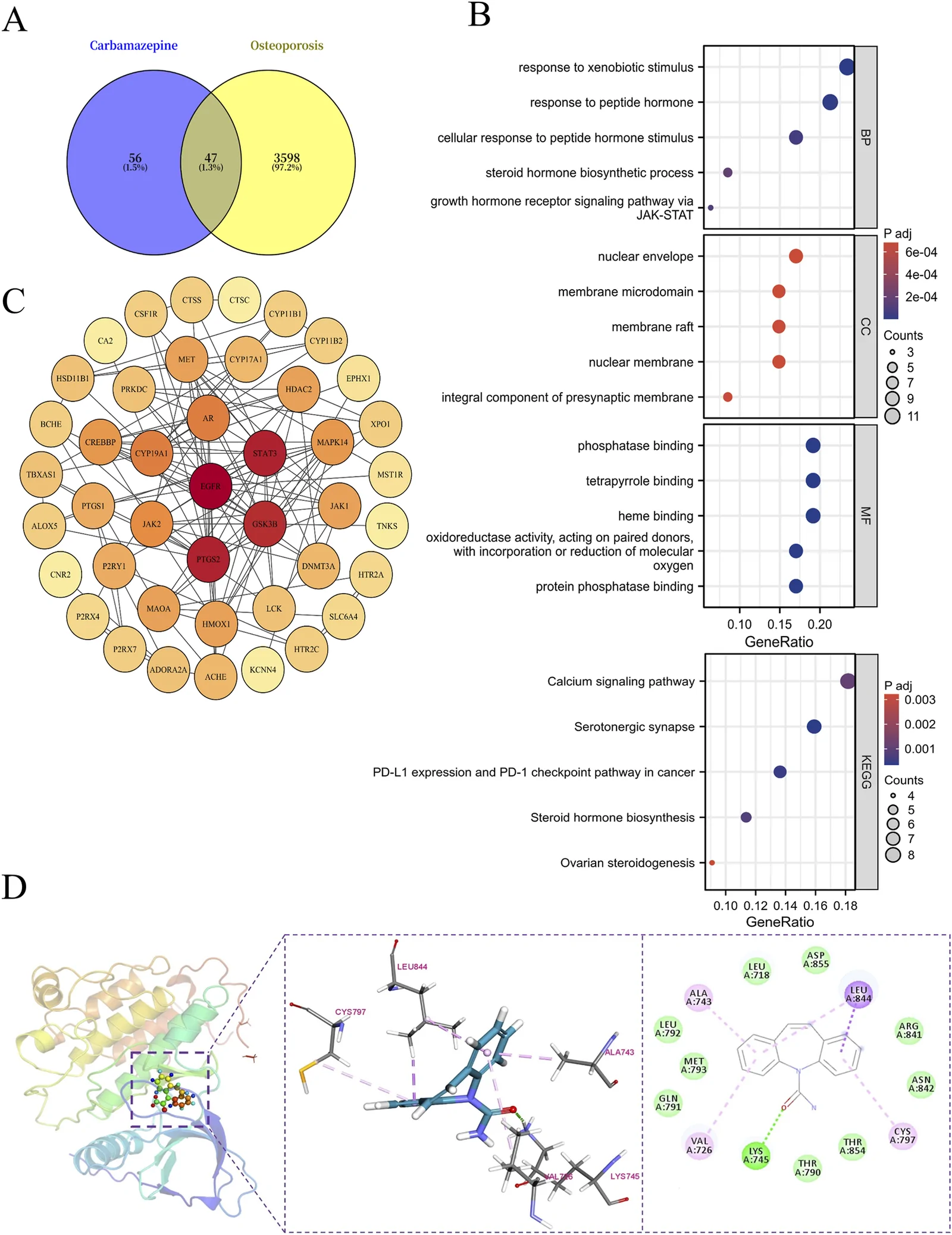
Target screening, functional enrichment analysis and molecular docking for carbamazepine underlying osteoporosis pathogenesis. **(A)** Venn diagram showing overlapping genes between carbamazepine targets and osteoporosis-associated genes. **(B)** GO (BP/CC/MF) and KEGG pathway enrichment analyses of intersecting targets. **(C)** PPI network of overlapping targets; node color gradient indicates degree centrality. **(D)** Molecular docking visualization of carbamazepine bound to EGFR.

Based on topological parameters of the PPI network, epidermal growth factor receptor (EGFR) was identified as the primary hub gene (Figure 2C). Molecular docking results exhibited a strong binding affinity between CBZ and EGFR, yielding a docking score of −8 kcal/mol. The complex was stabilized by hydrogen bonding and hydrophobic interactions with key residues in the ligand-binding pocket (Figure 2D). These findings elucidate the molecular binding mode of CBZ to EGFR and highlight its role as a central node connecting CBZ exposure to osteoblast dysfunction in osteoporosis.

### 3.2 Effects of phenobarbital on osteoporosis

An overlap analysis identified 50 PB candidate targets intersecting with osteoporosis-associated genes (Figure 3A). GO enrichment highlighted their predominant involvement in biological processes including steroid hormone response, oxidative stress, lipopolysaccharide response, and chemical stress response (Figure 3B). Molecular function enrichment emphasized nuclear receptor activity, nuclear steroid receptor activity, and metalloendopeptidase activity. KEGG enrichment revealed significant involvement in estrogen signaling, endocrine resistance, and TNF and IL-17 signaling pathways, which may contribute to PB-mediated abnormalities in bone metabolism.

**FIGURE 3:**
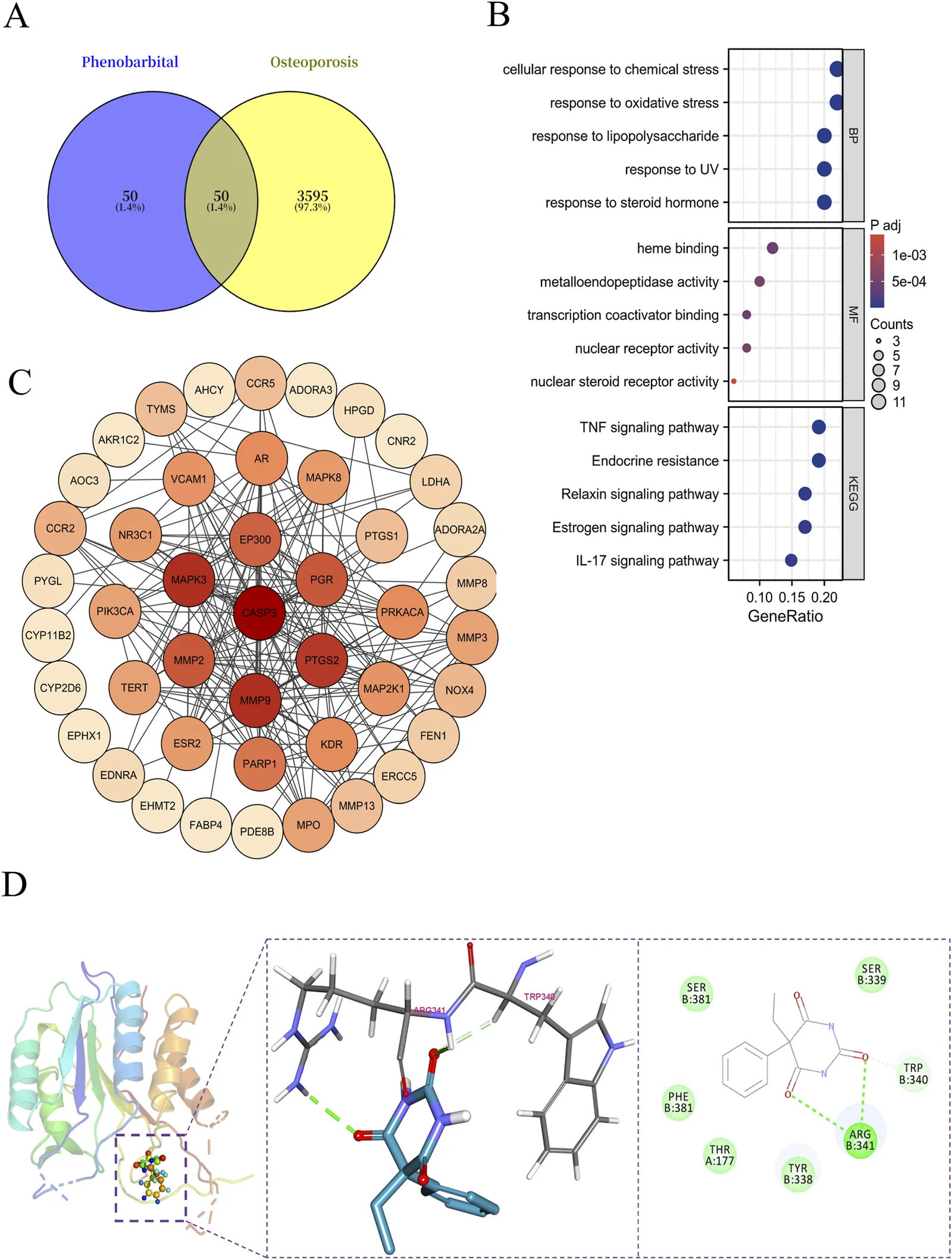
Target identification, enrichment analysis, and molecular docking of phenobarbital related to osteoporosis pathogenesis. **(A)** Venn diagram showing overlapping phenobarbital targets and osteoporosis-related genes. **(B)** GO and KEGG enrichment analyses of overlapping targets. **(C)** PPI network of overlapping targets, node color denotes degree centrality. **(D)** Molecular docking result of phenobarbital binding to CASP3.

Ranking based on three core topological indicators (degree, betweenness centrality, and closeness centrality) identified caspase-3 (CASP3) as the top hub gene within the PB-associated target network (Figure 3C). Molecular docking analysis yielded a docking score of −6.0 kcal/mol for the PB–CASP3 complex, indicating stable binding affinity. Hydrogen bonds and van der Waals forces formed with residues in the ligand-binding pocket collectively contribute to the conformational stability of the complex (Figure 3D). These computational predictions implicate CASP3 as a candidate hub target that may mediate PB-induced bone metabolic disorders.

### 3.3 Effects of phenytoin on osteoporosis

In the PHT group, 46 common targets were identified from the intersection of PHT targets and osteoporosis-related genes (Figure 4A). GO biological process enrichment analysis indicated that these targets were primarily involved in response to lipopolysaccharide, regulation of protein transport, serine phosphorylation, response to biological stimulus, and serine modification (Figure 4B). At the cellular component level, these targets displayed predominant localization in vesicular lumen, membrane rafts, and membrane microdomains, while molecular function enrichment highlighted significant roles in phosphatase binding, protein tyrosine kinase activity, heme binding, and protein serine/threonine kinase activity. KEGG pathway enrichment analysis revealed substantial involvement in the Rap1, ErbB, VEGF, and prolactin signaling pathways, suggesting their potential roles in PHT-triggered dysregulation of bone metabolism.

**FIGURE 4:**
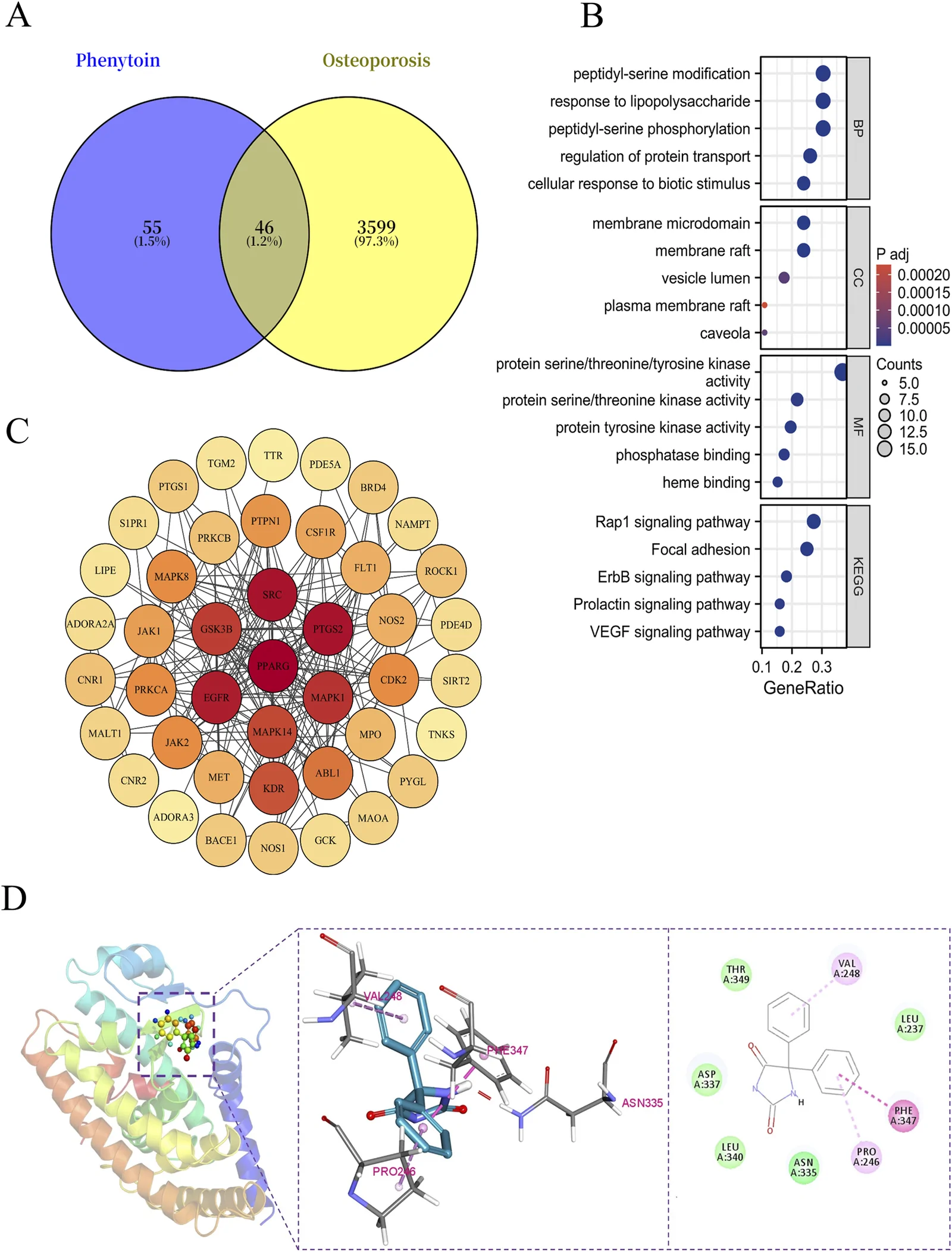
Target identification, enrichment analysis, and molecular docking of phenytoin related to osteoporosis pathogenesis. **(A)** Venn diagram showing overlapping phenytoin targets and osteoporosis-related genes. **(B)** GO and KEGG enrichment analyses of overlapping targets. **(C)** PPI network of overlapping targets, node color denotes degree centrality. **(D)** Molecular docking result of phenytoin binding to PPARG.

Topological parameter analysis, encompassing degree, betweenness centrality, and closeness centrality, identified peroxisome proliferator-activated receptor gamma (PPARG) as the primary hub gene within the PHT target network (Figure 4C). Molecular docking results demonstrated a strong binding affinity between PHT and PPARG, with a docking score of −7.4 kcal/mol. Multiple noncovalent interactions, including π–π stacking, π-alkyl contacts, and van der Waals forces with key residues in the ligand-binding pocket, contributed to the structural stability of the PHT-PPARG complex (Figure 4D). These computational predictions position PPARG as a candidate hub target potentially involved in PHT-associated effects on osteoblast function.

### 3.4 Effects of valproic acid on osteoporosis

For VPA, Venn overlap analysis identified 48 overlapping targets shared with osteoporosis-related genes (Figure 5A). GO enrichment analysis indicated that these targets primarily participated in biological processes such as steroid metabolic processes, steroid biosynthesis, and peptide hormone response (Figure 5B), with molecular function enrichment dominated by steroid dehydrogenase activity. KEGG pathway enrichment analysis further highlighted significant involvement in the regulation of lipolysis in adipocytes and neuroactive ligand-receptor interaction pathways, which may contribute to VPA-induced dysregulation of bone metabolism.

**FIGURE 5:**
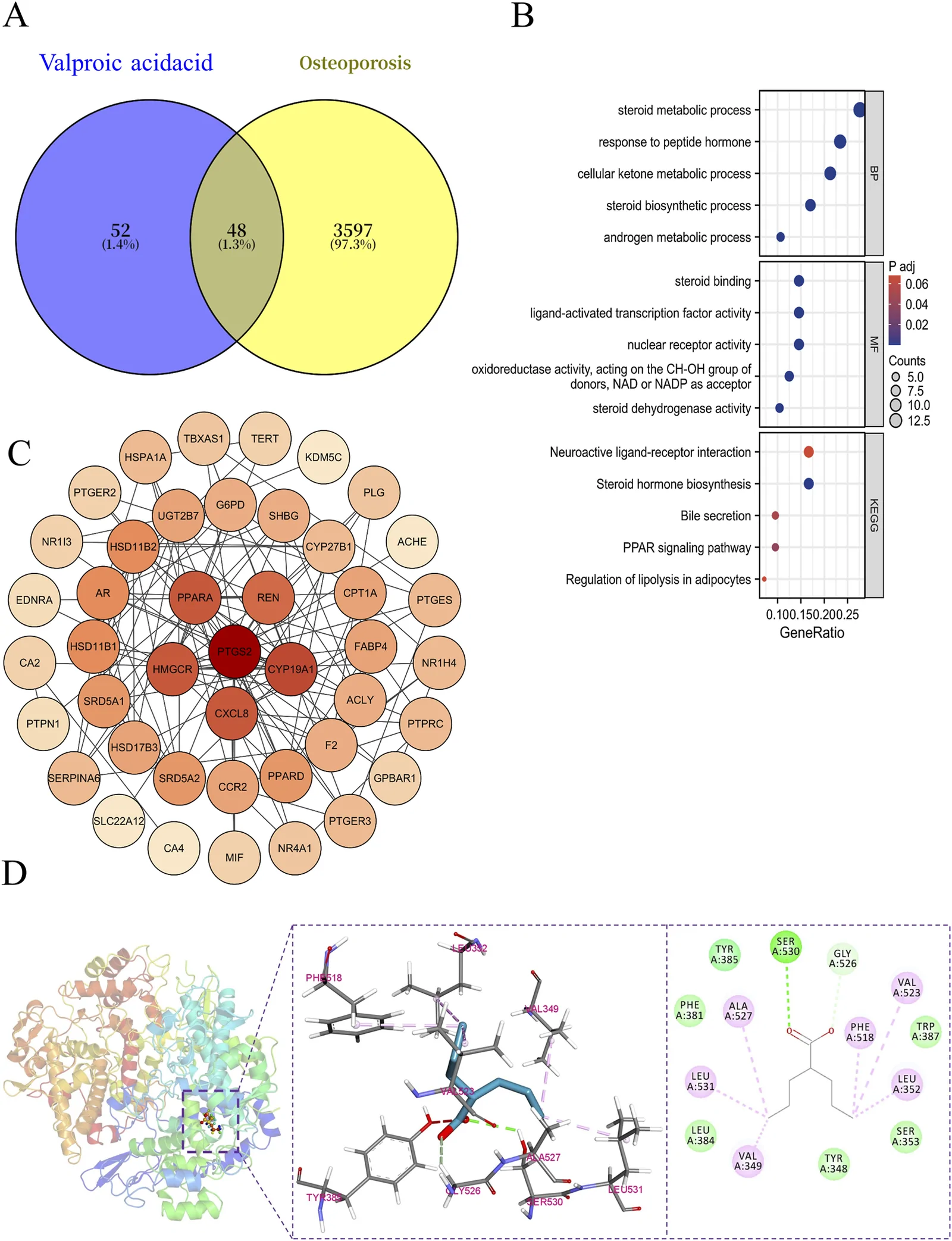
Target identification, enrichment analysis, and molecular docking of Valproic acid related to osteoporosis pathogenesis. **(A)** Venn diagram showing overlapping Valproic acid and osteoporosis-related target genes. **(B)** GO and KEGG enrichment analyses of intersecting targets. **(C)** PPI network of overlapping targets, node color denotes degree centrality. **(D)** Molecular docking result of Valproic acid binding to PTGS2.

Analysis of topological features within the PPI network revealed prostaglandin-endoperoxide synthase 2 (PTGS2) as the most prominent hub gene (Figure 5C). Molecular docking assays yielded a docking score of −5.9 kcal/mol between VPA and PTGS2, indicating moderate binding affinity between the ligand and the protein. Conventional hydrogen bonds formed with key residues in the ligand-binding pocket served as the primary force maintaining the structural stability of the complex (Figure 5D). These computational predictions suggest PTGS2 as a candidate hub target that may be involved in VPA-associated effects on osteoblast function, pending experimental validatio.

### 3.5 Molecular dynamics simulation

All-atom MD simulations of 100 ns duration were conducted to evaluate the dynamic behavior of four AED–target complexes: CBZ–EGFR, PB–CASP3, PHT–PPARG, and VPA–PTGS2. To minimize reliance on visual inspection alone, quantitative trajectory analyses were performed over the equilibrated 50–100 ns interval, with values reported as mean ± SD in Supplementary Table S9.

RMSD analysis (Figure 6) was utilized to assess conformational stability post-equilibration. During the 50–100 ns interval, PB-CASP3, PHT-PPARG, and VPA-PTGS2 displayed relatively stable complex RMSD values of 0.22 ± 0.02, 0.24 ± 0.02, and 0.18 ± 0.01 nm, respectively. Corresponding protein RMSD values were 0.13 ± 0.03, 0.17 ± 0.02, and 0.14 ± 0.01 nm, indicating stable protein backbone behavior throughout the equilibrated phase. In contrast, CBZ-EGFR exhibited a higher complex RMSD of 0.34 ± 0.02 nm and a protein RMSD of 0.27 ± 0.03 nm, suggesting greater conformational flexibility of EGFR upon CBZ binding rather than a simple loss of ligand retention. RMSF analysis further supported this interpretation, with CBZ-EGFR showing the highest average RMSF among the four systems (0.15 ± 0.11 nm), while PB-CASP3, PHT-PPARG, and VPA-PTGS2 presented lower average RMSF values of 0.11 ± 0.07, 0.12 ± 0.07, and 0.11 ± 0.05 nm, respectively.

**FIGURE 6:**
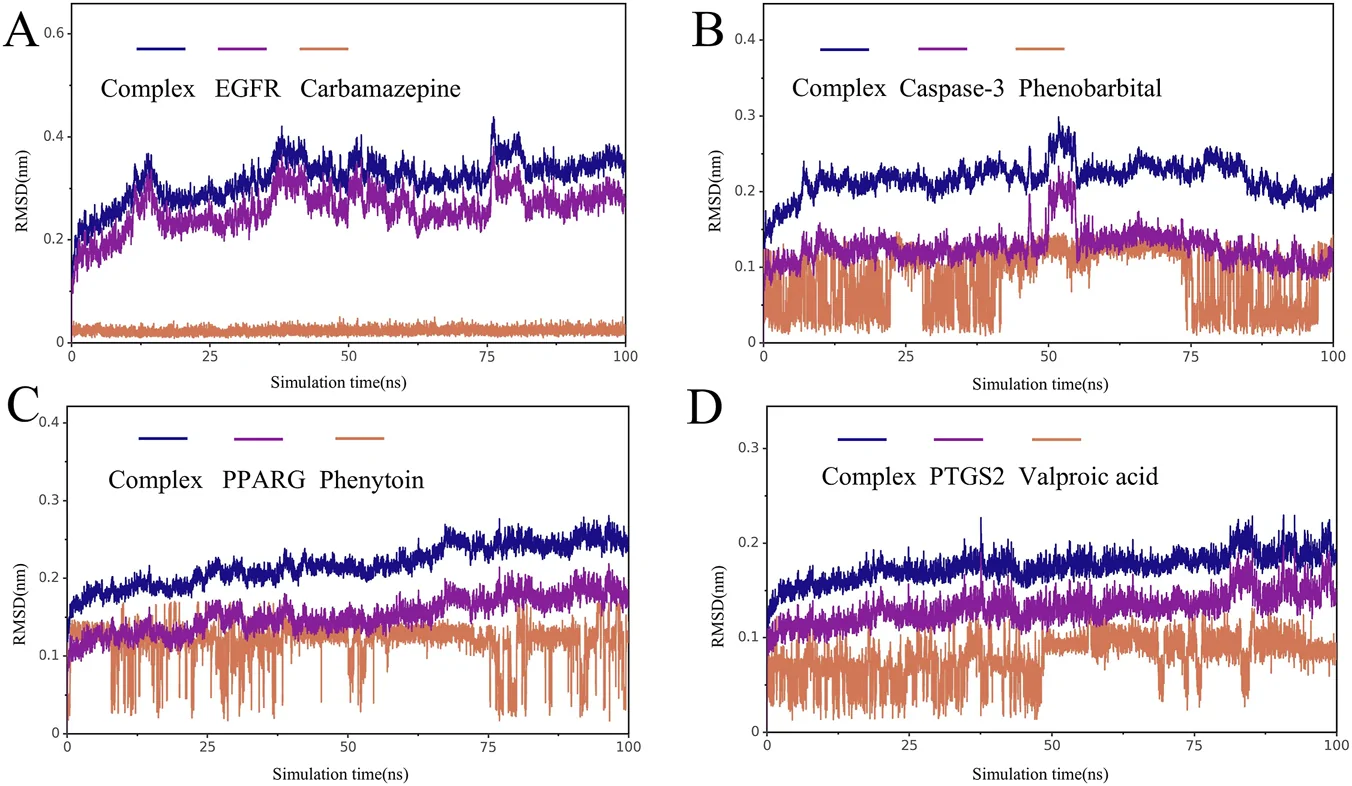
RMSD profiles derived from 100 ns molecular dynamics simulations of four AED-target complexes. **(A)** EGFR-carbamazepine complex **(B)** CASP3-phenobarbital complex **(C)** PPARG-phenytoin complex **(D)** PTGS2-Valproic acid complex. Blue: complex RMSD; purple: protein backbone RMSD; orange: ligand RMSD.

Ligand RMSD and distance metrics were analyzed to assess ligand retention and positional stability. During the equilibrated phase, ligand RMSD values remained low: 0.09 ± 0.04 nm for PB in CASP3, 0.12 ± 0.03 nm for PHT in PPARG, 0.09 ± 0.01 nm for VPA in PTGS2, and 0.02 ± 0.01 nm for CBZ in EGFR. Protein–ligand distance analysis yielded average distances of 2.26 ± 0.14 nm for PB–CASP3, 2.19 ± 0.05 nm for PHT–PPARG, 2.35 ± 0.04 nm for VPA-PTGS2, and 1.50 ± 0.04 nm for CBZ-EGFR. The corresponding docking-site-ligand distances were 1.16 ± 0.25, 0.66 ± 0.04, 2.28 ± 0.03, and 0.75 ± 0.02 nm, respectively. These findings indicate that the ligands remained associated with their target proteins throughout the equilibrated trajectory segments, albeit with system-specific positional patterns. Notably, VPA-PTGS2 exhibited a larger docking-site-ligand distance than the other systems, suggesting a more displaced ligand position relative to the predicted docking site.

Rg (Figure 7) and SASA (Figure 8) were employed to evaluate structural compactness and solvent exposure. Absolute Rg and SASA values, influenced by protein size and oligomeric state, were interpreted within each protein–ligand system rather than compared directly across different targets. Rg values exhibited minimal fluctuations during the 50–100 ns interval: 1.77 ± 0.01 nm for PB-CASP3, 1.92 ± 0.01 nm for PHT-PPARG, 3.17 ± 0.01 nm for VPA-PTGS2, and 2.04 ± 0.01 nm for CBZ-EGFR. Complex SASA values also remained stable, with measurements of 118.57 ± 2.02, 144.65 ± 2.25, 440.22 ± 3.69, and 163.30 ± 2.51 nm^2^ for PB-CASP3, PHT-PPARG, VPA-PTGS2, and CBZ-EGFR, respectively. These results indicate that no substantial global expansion or collapse occurred during the equilibrated phase.

**FIGURE 7:**
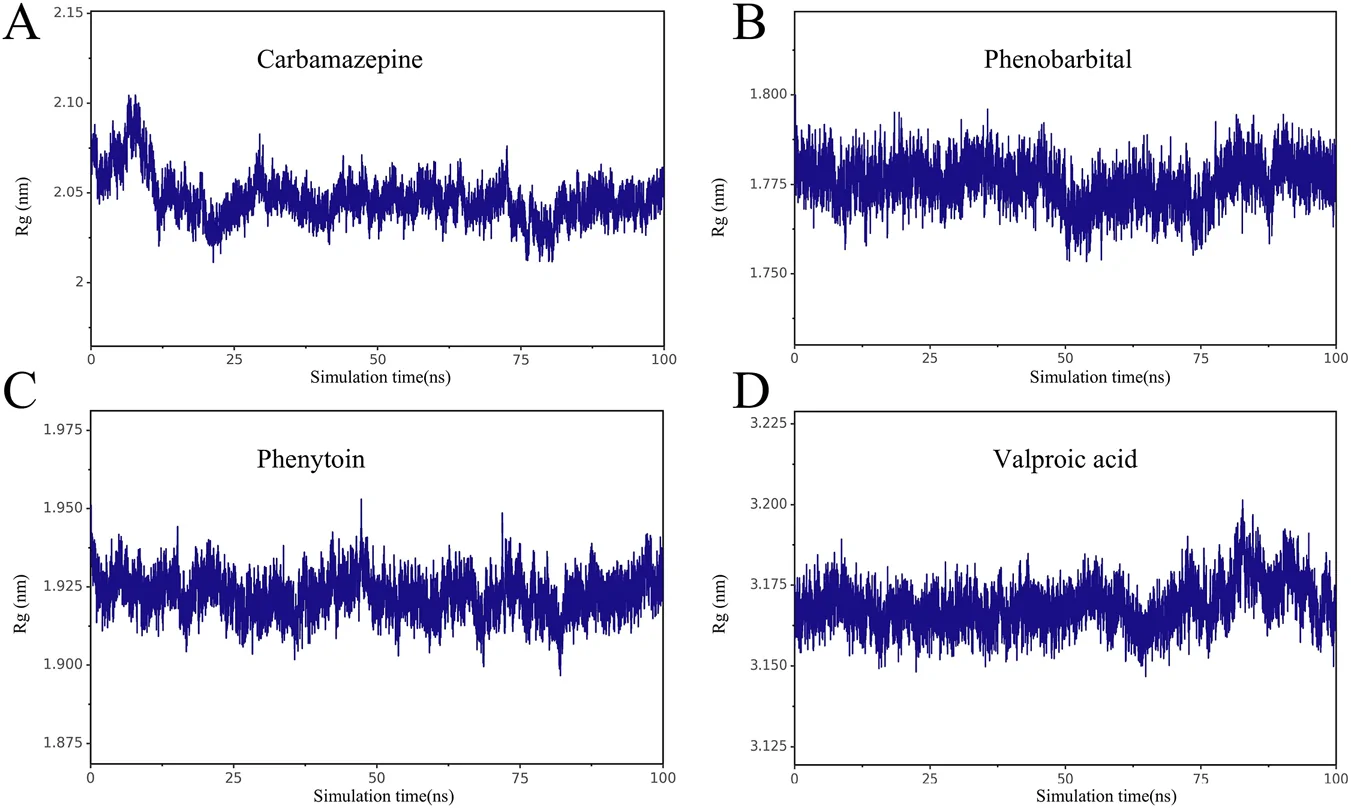
Radius of gyration (Rg) profiles from 100 ns molecular dynamics simulations of four AED-target complexes. **(A)** EGFR-carbamazepine **(B)** CASP3-phenobarbital **(C)** PPARG-phenytoin **(D)** PTGS2-Valproic acid.

**FIGURE 8:**
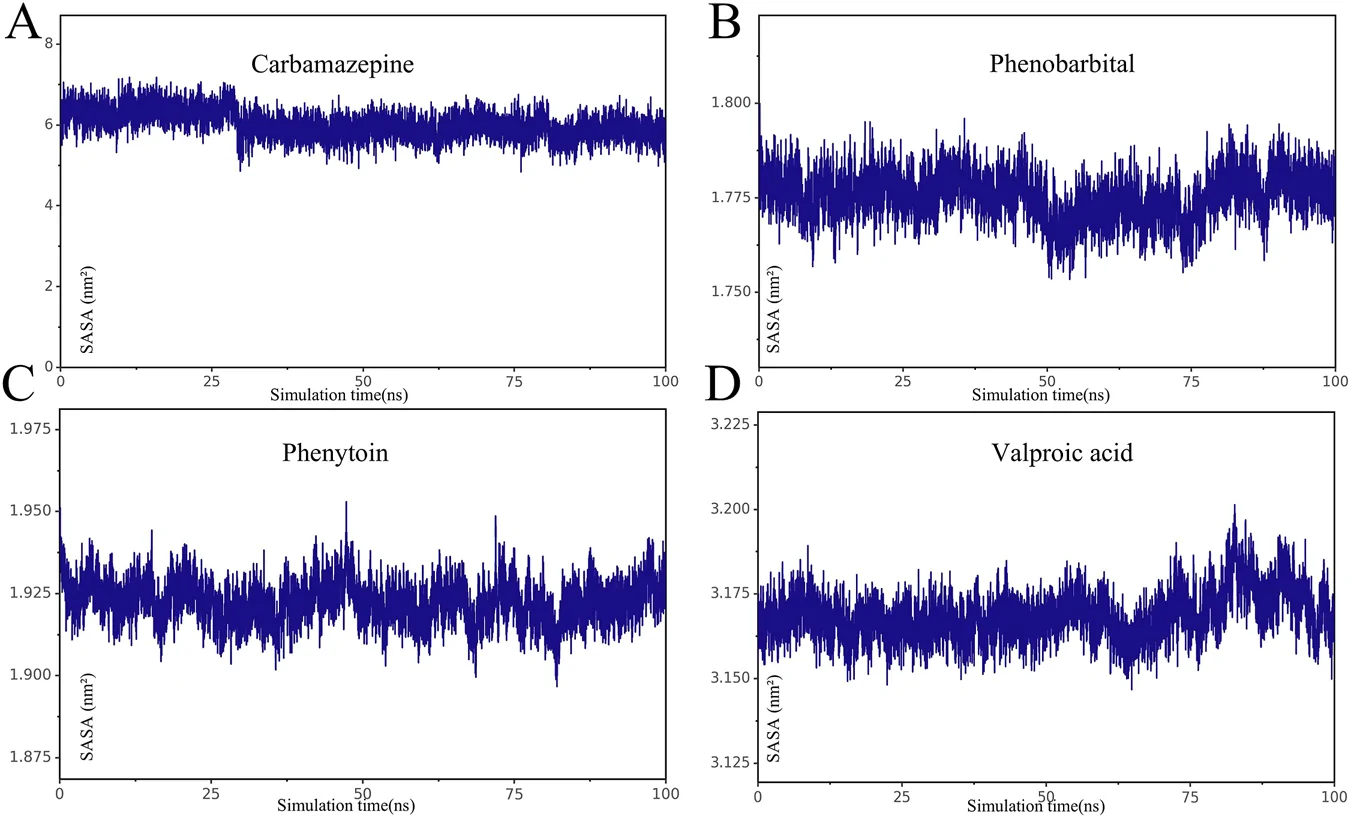
Solvent-accessible surface area (SASA) profiles from 100 ns molecular dynamics simulations of four AED-target complexes. **(A)** EGFR-carbamazepine **(B)** CASP3-phenobarbital **(C)** PPARG-phenytoin **(D)** PTGS2-Valproic acid.

Hydrogen-bond analysis (Figure 9) revealed ligand-specific interaction patterns. During the 50–100 ns interval, the average hydrogen-bond numbers were 0.82 ± 0.59 for PB-CASP3, 1.61 ± 0.86 for PHT-PPARG, 2.09 ± 0.44 for VPA-PTGS2, and 0.98 ± 0.14 for CBZ-EGFR. These differences reflect distinct polar interaction profiles rather than serving as direct measures of comparative binding strength across chemically heterogeneous ligands.

**FIGURE 9:**
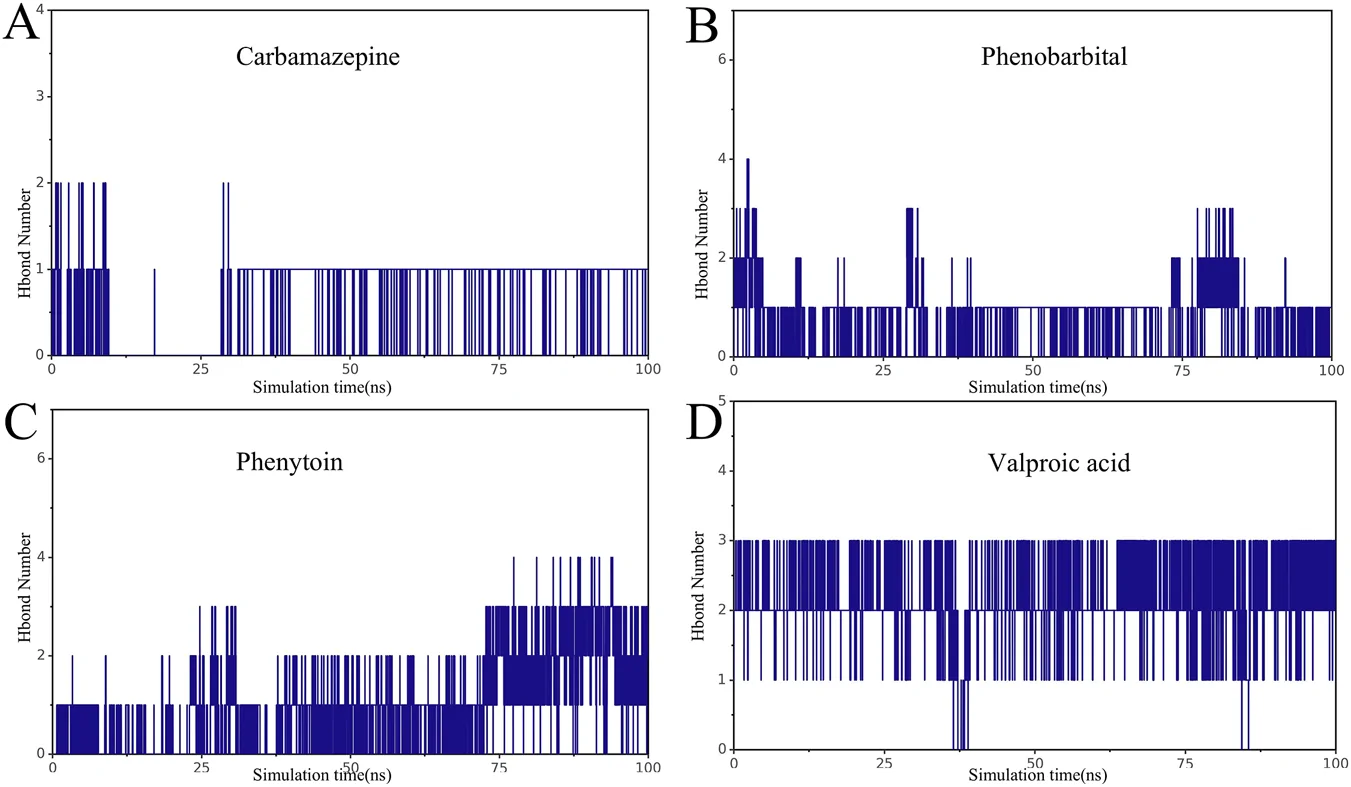
Time-evolution of hydrogen bond counts in 100-ns MD simulations for four AED–target complexes. **(A)** EGFR-carbamazepine **(B)** CASP3-phenobarbital **(C)** PPARG-phenytoin **(D)** PTGS2-Valproic acid.

MM-PBSA calculations (Supplementary Table S9) were performed using snapshots extracted from the equilibrated 50–100 ns interval to provide supportive energetic evidence for the docking and MD results. The CBZ–EGFR complex exhibited the most favorable calculated binding free energy, with ΔG_bind = −85.986 ± 0.843 kJ/mol, followed by PB–CASP3 (−49.342 ± 5.205 kJ/mol), PHT–PPARG (−26.474 ± 2.533 kJ/mol), and VPA–PTGS2 (−12.260 ± 1.359 kJ/mol). Van der Waals interactions contributed favorably in all systems, while polar solvation energy partially offset the favorable molecular mechanics terms.

Notably, MM-PBSA calculations do not explicitly account for configurational entropy and are subject to continuum-solvent approximations, particularly in the polar solvation term. Hence, these binding free-energy values should be interpreted as relative supportive indicators rather than absolute experimental binding affinities.

Supplementary Figure 1 displays superimposed structural snapshots of all four complexes extracted from equilibrated 50–100 ns trajectories. Snapshots were sampled every 10 ns and superimposed in PyMOL via protein backbone alignment, with panels A–D representing PB-CASP3, PHT-PPARG, VPA-PTGS2, and CBZ-EGFR, sequentially. While Supplementary Figure 1 provides a global view of conformational fluctuation and ligand positional drift, comprehensive quantitative trajectory metrics—including time-series RMSD, RMSF, Rg, buried SASA, protein-ligand distance, decomposed binding energy, residue-wise energy contribution, dynamic hydrogen bond count, and atomic-level binding interaction graphs for each system—are available in Supplementary Figures 2–5, respectively. These structural overlays should be interpreted alongside the quantitative ligand RMSD, interatomic distance, hydrogen bond, and MM-PBSA energetic data. Notably, trajectory clustering and residue contact occupancy analyses were not conducted; therefore, the superimposed snapshots do not represent cluster centroids or characteristic residue contact states.

In summary, quantitative MD analysis, MM-PBSA calculations, and superimposed structural snapshots indicate that the four AED–target complexes exhibit distinct dynamic and energetic profiles. PB–CASP3, PHT–PPARG, and VPA–PTGS2 maintained stable structural metrics during the equilibrated phase, whereas CBZ–EGFR displayed greater protein conformational flexibility along with the most favorable calculated MM-PBSA binding free energy. These results should be regarded as supportive dynamic and energetic evidence rather than definitive proof of comparative binding strength or experimentally validated binding affinity.

### 3.6 Effects of AEDs on the viability of MC3T3-E1 osteoblasts

The CCK-8 assay was conducted to evaluate the cytotoxic effects of CBZ, PB, PHT, and VPA on MC3T3-E1 osteoblasts after 24 h and 48 h of treatment. Following exposure to serially diluted AEDs, relative cell viability was normalized to the DMSO vehicle control group. Each experimental group contained three technical replicate wells, and all experiments were conducted with three independent biological replicates to ensure reliability. No significant cytotoxic phenotype was observed at the selected concentration gradients of CBZ (20, 40 μM), PB (20, 40 μM), PHT (10, 20 μM), and VPA (500, 800 μM) at either 24 h or 48 h, with the relative viability of drug-treated cells remaining close to 100% compared to vehicle controls (Supplementary Table S10; Supplementary Figure S6). These findings indicate that the working concentrations used in subsequent functional experiments did not induce notable acute cytotoxicity or short-term proliferative suppression in MC3T3-E1 cells within 48 h.

### 3.7 AEDs inhibit clonogenic ability of MC3T3-E1 cells

Colony formation assays demonstrated that all four tested AEDs significantly suppressed the clonogenic potential of MC3T3-E1 osteoblasts. Both the quantity and size of cell colonies were markedly reduced following drug treatment (*p* < 0.05), indicating persistent impairment of clonogenic capacity upon AED exposure (Figure 10; Supplementary Table S11).

**FIGURE 10:**
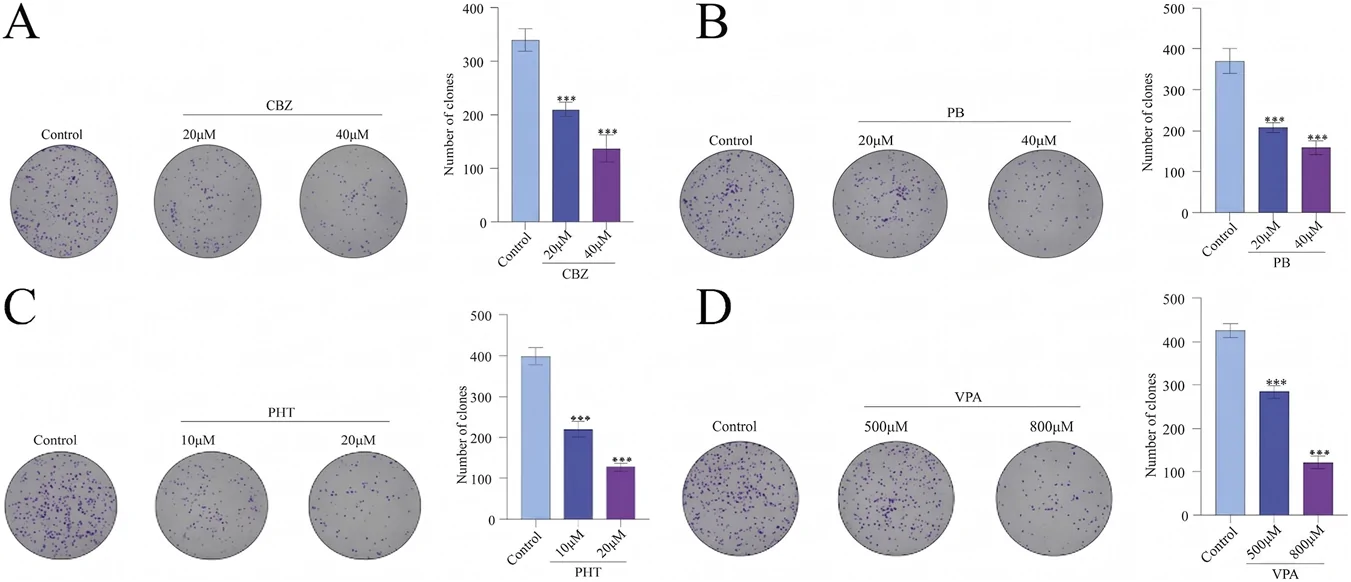
Four AEDs suppress colony formation of MC3T3-E1 osteoblasts. **(A–D)** Quantification of colony counts after treatment with gradient concentrations of CBZ, PB, PHT and VPA separately. ***p < 0.05 versus control group.

### 3.8 AEDs regulate the expression of core hub proteins

Western blot analysis was performed to investigate the regulatory effects of AEDs on the expression of key target proteins in MC3T3-E1 osteoblasts (Figure 11). After 48 h of drug intervention, 40 μM CBZ significantly downregulated p-EGFR expression, while total EGFR protein levels remained unchanged. In contrast, 40 μM PB resulted in a significant increase in cleaved caspase-3 abundance with no observable change in total caspase-3, indicating the proteolytic activation of caspase-3. Furthermore, 20 μM PHT substantially upregulated PPARG expression, and 800 μM VPA significantly increased PTGS2 protein levels. Raw grayscale values for each biological replicate and corresponding blot images are summarized in Supplementary Table S12. Full uncropped Western blot scans for all independent replicates are available in Supplementary File 2.

**FIGURE 11:**
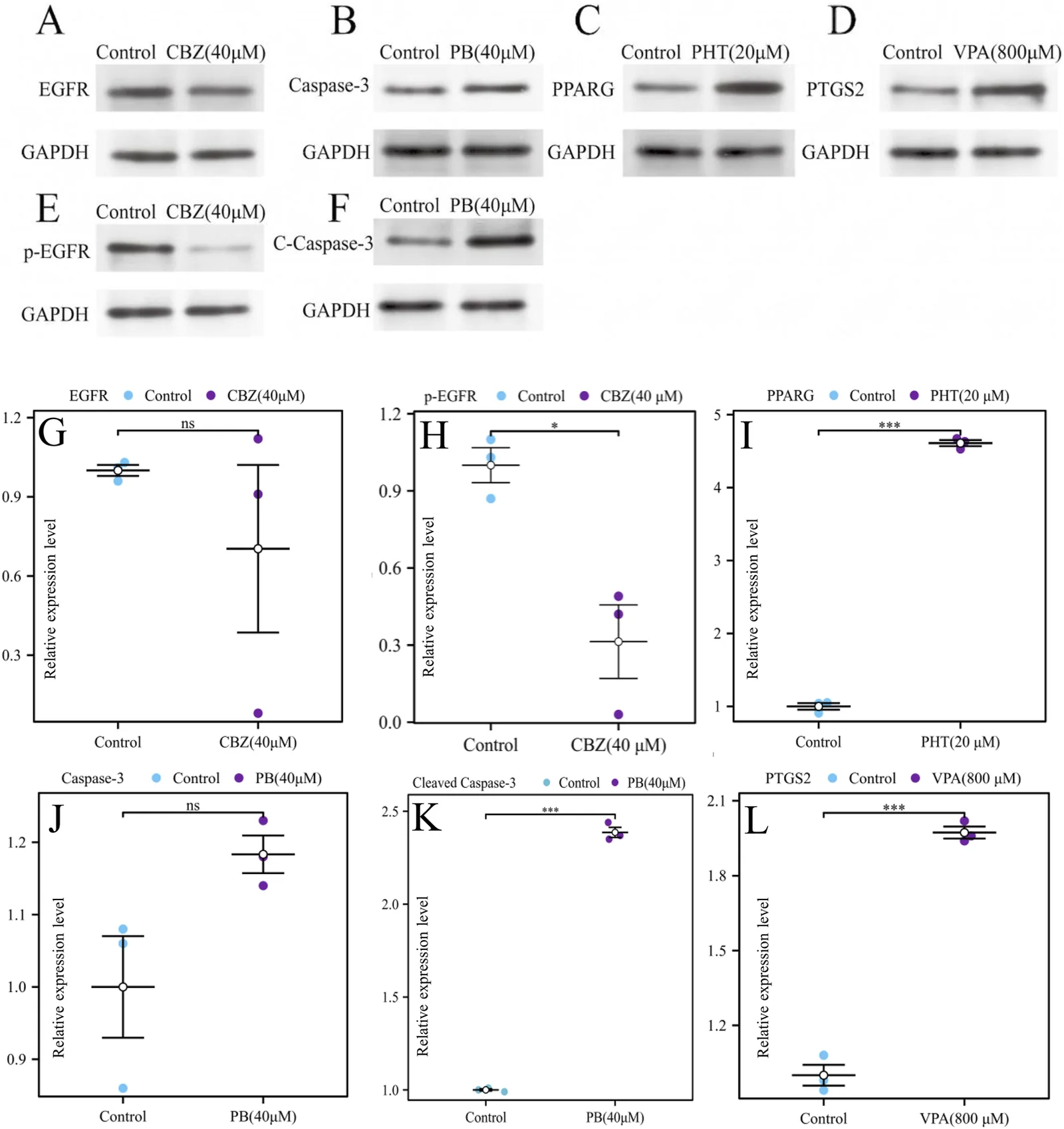
Effects of four antiepileptic drugs on key target protein expression. **(A–F)** Western blot bands of EGFR, phosphorylated EGFR (p-EGFR), Caspase-3, cleaved Caspase-3 (C-Caspase-3), PPARG and PTGS2 after treatment with CBZ (40 μM), PB (40 μM), PHT (20 μM) and VPA (800 μM); GAPDH served as loading control. **(G–L)** Grayscale quantification of relative protein expression. Data are shown as mean ± SD; ***p < 0.05 versus control group.

## 4 Discussion

Long-term use of AEDs represents a significant risk factor for secondary osteoporosis; however, the underlying molecular mechanisms remain insufficiently elucidated. Extensive research has established robust associations between abnormal steroid hormone metabolism, increased oxidative stress responses, and the onset and progression of osteoporosis (Williams et al., 2019). Steroid hormone imbalances disrupt the delicate equilibrium between osteoblast-mediated bone formation and osteoclast-driven bone resorption (Zhang et al., 2026), while elevated intracellular reactive oxygen species (ROS) levels induce persistent oxidative stress that accelerates bone matrix degradation and diminishes bone mineral density (Feng and Tang, 2014; Wang et al., 2024). These interconnected pathophysiological processes form a critical molecular basis for osteoporosis progression and provide a theoretical foundation for the development of clinical prevention strategies.

Aligned with these established mechanisms, GO and KEGG enrichment analyses demonstrated that the shared targets of the four AEDs were predominantly enriched in steroid hormone biosynthesis, estrogen signaling, and oxidative stress-related pathways, thereby highlighting the involvement of these processes in AED-induced bone loss. Furthermore, significant enrichment was observed in processes regulating peptide response and trans-synaptic signaling, suggesting that dysregulation of neuroendocrine and synaptic communication may further contribute to the pathogenesis of AED-related osteoporosis. Moving beyond traditional studies focused on single AEDs or descriptive clinical associations, this study integrates network toxicology, molecular docking, MD simulations, and *in vitro* cell experiments to systematically explore the mechanisms of osteoporosis induction by four classic AEDs. Through multi-database mining, protein–protein interaction network construction, and topological analysis, EGFR, CASP3, PPARG, and PTGS2 were identified as core hub genes, thereby refining specific functional targets. Molecular docking suggested favorable binding affinities for these targets, while quantitative MD simulations and MM-PBSA calculations provided supportive evidence for distinct dynamic and energetic behaviors of the corresponding complexes. Together with *in vitro* validation, these findings establish a structural framework for interpreting the observed experimental phenotypes.

EGFR is pivotal in signaling pathways that regulate osteoblast proliferation, differentiation, and survival; its dysregulation disrupts the balance between bone formation and resorption (Schneider et al., 2009). Although molecular docking predicted a favorable binding affinity (−8 kcal/mol) between CBZ and EGFR, quantitative MD analysis revealed greater conformational flexibility of the CBZ–EGFR complex compared to other systems. However, ligand RMSD and distance metrics indicated that CBZ remained within or close to the predicted binding region during the equilibrated trajectory segment, and MM-PBSA analysis demonstrated the most favorable calculated binding free energy among the four complexes. These results suggest an adaptive binding pattern mediated by EGFR conformational rearrangement, rather than weak binding stability or spontaneous ligand dissociation. Such dynamic binding behavior may disrupt normal EGFR activation dynamics. Reduced expression of phosphorylated EGFR (p-EGFR) with unchanged total receptor protein levels further supports this notion, confirming that CBZ treatment may exert functional regulation over EGFR signaling pathways.

CASP3, a key executioner caspase, mediates osteoblast and osteocyte apoptosis, processes vital to bone remodeling and turnover (Cerri et al., 2026). Research on the anti-osteoporotic effects of traditional Chinese medicine has identified CASP3 as a pivotal target (Li et al., 2023), while network pharmacology analysis of notoginsenoside R1 also highlighted CASP3 as a core factor associated with apoptosis and bone metabolism (Chen et al., 2026). These findings suggest that CASP3 may function as a central molecular hub linking AED exposure to osteoblast dysfunction and apoptosis (Dilber et al., 2025). MD simulations revealed that the PB–CASP3 complex maintained overall conformational equilibrium, with intermittent hydrogen-bond formation and favorable MM-PBSA binding free energy. RMSF profiles indicated that ligand binding significantly increased the flexibility of distal loop regions while the binding pocket remained relatively stable, suggesting that PB binding may induce localized dynamic changes in CASP3. Corresponding to this computational profile, Western blot analysis demonstrated a marked increase in cleaved-CASP3 levels without a corresponding rise in total CASP3, indicating heightened CASP3 processing. Notably, activation of CASP3 cleavage, whether through the extrinsic or intrinsic pathway, requires interaction with other proteins (Pecina et al., 2010; Neumann et al., 2010).

PPARG, a nuclear receptor transcription factor expressed in osteoblasts and osteocytes, critically modulates the balance between bone formation and resorption (Baroi et al., 2021). Accumulating evidence confirms that PPARG activation drives adipogenic differentiation of bone marrow-derived mesenchymal stem cells while repressing osteogenic differentiation (Akune et al., 2004), thereby serving as a key molecular switch governing osteogenic-adipogenic lineage commitment (Lecka-Czernik et al., 1999). Valproic acid suppresses PPARG signaling in neuronal and adipogenic models (Lan et al., 2008; Yuyama and Fujimori, 2014); however, its effects on PPARG in osteocytes remain elusive. Molecular docking identified PPARG as a high-affinity binding target of PHT, with a binding free energy of −7.4 kcal/mol. Subsequent MD simulations revealed stable structural features of the PHT–PPARG complex at equilibrium, including low RMSD for both the protein and ligand and consistent distances between PHT and the binding pocket. The average number of hydrogen bonds reflected specific polar interactions between the ligand and receptor, with MM-PBSA calculations further validating this association. Nonetheless, these computational results support stable ligand–receptor interactions and favorable dynamic compatibility but do not directly demonstrate PPARG activation. Consistent with the *in silico* evidence, Western blot results indicated markedly elevated PPARG protein levels following PHT intervention.

PTGS2 encodes cyclooxygenase-2 (COX-2), a key enzyme in prostaglandin synthesis that plays a pivotal regulatory role in bone remodeling by modulating osteoblast and osteoclast activity (Schroeder and Westendorf, 2005). As a classic histone deacetylase inhibitor, VPA regulates the expression of multiple genes through epigenetic modification. Previous *in vitro* studies indicate that VPA induces apoptosis by upregulating COX-2 expression, with this pro-apoptotic effect reversible by the specific COX-2 inhibitor NS-398 (Fan et al., 2020). Consistent with these findings, molecular docking and MD simulation analyses suggested that the VPA–PTGS2 complex maintained stable structural metrics during the equilibrated trajectory segment and exhibited the highest average hydrogen-bond number among the four systems. However, MM-PBSA analysis yielded the weakest calculated binding free energy for VPA–PTGS2, indicating that frequent polar contacts do not necessarily correlate with the strongest overall energetic favorability. Consequently, the experimentally observed PTGS2 upregulation should not be attributed solely to direct binding. The underlying mechanism of PTGS2 upregulation—whether through direct protein stabilization, altered degradation, or VPA’s established epigenetic transcriptional regulation—remains to be experimentally elucidated. Furthermore, colony formation assays demonstrated that VPA significantly reduced the number and size of MC3T3-E1 cell colonies.

The distinct molecular interaction patterns identified in this study offer valuable insights for assessing bone-related risks and tailoring AED selection for patients undergoing long-term seizure management. First, the current data indicate that bone health monitoring should vary according to different AED regimens. For example, CBZ demonstrates a high binding affinity toward EGFR, as supported by favorable MM-PBSA binding energy and notable conformational flexibility observed in MD simulations. These findings suggest that patients receiving long-term CBZ treatment may exhibit increased skeletal vulnerability. Second, the four AEDs investigated induce osteoporotic alterations through distinct molecular targets and downstream signaling pathways, establishing a mechanistic foundation for personalized AED administration. In the present study, we found that VPA upregulates PTGS2 expression, which may contribute to osteoporosis-related phenotypes. Based on these results, co-administration of low-dose COX-2 inhibitors could alleviate AED-induced bone loss. Nevertheless, support for this inference is limited to computational and *in vitro* data, with no human *in vivo* evidence. Therefore, further studies using cellular and animal models are required to validate this potential protective effect. Moreover, large-scale prospective clinical cohort investigations are needed to assess whether these preliminary computational findings can be translated into clinical practice.

This study presents several notable limitations. First, target prediction, pathway enrichment, and molecular docking assessments were entirely computational. While core predicted targets were validated through Western blot analyses in MC3T3-E1 osteoblasts, functional verification was limited to a single osteoblast cell line, with no *in vivo* animal models utilized that more accurately replicate human bone metabolism. Second, only one independent 100-ns MD trajectory was run for each protein–ligand complex. Although trajectory properties were quantified and MM-PBSA binding free energies computed, replicate simulations with distinct initial atomic velocities were not conducted. Extended simulations with multiple repeated trajectories are necessary to validate the reliability of the molecular interaction signatures observed. Additionally, this study did not quantify residue–residue contact occupancy or the precise distances between ligands and key binding residues. Third, the screening focused solely on four classic AEDs, without incorporating newer antiepileptic agents, limiting the generalizability of the findings. Consequently, the identified molecular mechanisms may not be directly extrapolated to patients using novel AEDs in clinical practice. Despite these limitations, this study offers preliminary insights into the core molecular mechanisms of AED-induced osteoporosis, providing evidence to support evidence-based clinical decision-making and skeletal health management for patients with epilepsy.

## 5 Conclusion

This study preliminarily investigated potential molecular mechanisms related to AED-induced osteoporosis using an integrated *in silico* workflow that combined network toxicology, molecular docking, and MD simulations, complemented by *in vitro* assessments. EGFR, CASP3, PPARG, and PTGS2 were identified as potential target regulators that may exert distinct modulatory effects on bone metabolism and osteoporosis progression. MD simulations revealed differing binding patterns across various drug–target complexes. Furthermore, Western blot analyses in MC3T3-E1 osteoblasts provided *in vitro* evidence supporting potential target-level alterations induced by the four conventional AEDs. Collectively, these computational and cellular observations offer preliminary mechanistic insights into understanding the differential osteogenic responses to AED treatment. However, these *in silico* predictions and limited *in vitro* findings require extensive validation in animal models and clinical cohorts to clarify their true translational relevance regarding AED-induced adverse bone effects.

## Data Availability

The original contributions presented in the study are included in the article/Supplementary Material, further inquiries can be directed to the corresponding author.
