# Supplementary material for: Insights into osteoporosis triggered by common antiepileptic drugs via network toxicology, molecular docking and molecular dynamics simulations
Source: Front Pharmacol. 2026 Sep 25;17:1908344. doi: 10.3389/fphar.2026.1908344 (PMC13649964; doi:10.3389/fphar.2026.1908344)
Supplement: Supplementary file 1 [file DataSheet1.DOCX]

Supplementary Material

# 1 Supplementary Files

## 1.1 S1：Original molecular docking source files for the four drug–target complexes

## 1.2 S2：Full Uncropped Western Blot Independent Replicates

# 2 Supplementary Figures and Tables

For more information on Supplementary Material and for details on the different file types accepted, please see [here](https://www.frontiersin.org/guidelines/author-guidelines#supplementary-material).

## 2. Supplementary Figures

**Supplementary Figure 1.** Superimposed structural snapshots of AED–target complexes extracted from the equilibrated 50–100 ns MD trajectory segments.Snapshots were extracted at 10-ns intervals from the equilibrated trajectory segments and superimposed in PyMOL by aligning the protein backbone. Panels show (A) PB–CASP3, (B) PHT–PPARG, (C) VPA–PTGS2, and (D) CBZ–EGFR. Proteins are shown as cartoons, and ligands are shown as sticks. The superimposed snapshots provide a structural visualization of ligand positional behavior relative to the predicted binding regions and should be interpreted together with quantitative ligand RMSD, distance metrics, hydrogen-bond number, and MM-PBSA results. These snapshots were not generated by RMSD-based clustering and should not be interpreted as cluster centroids or residue-level contact-occupancy evidence.

**Supplementary Figure 2**. Molecular dynamics simulation analysis of EGFR-carbamazepine complex Subgraph description：(A) RMSD of complex, protein and ligand; (B) Rg of protein; (C) RMSF of each residue; (D) average distance between pocket-ligand and protein-ligand; (E) buried SASA; (F) protein conformational clustering; (G) binding energy decomposed into electrostatic (ELE) and van der Waals (VDW) components; (H) residue-wise binding energy contribution; (I) hydrogen bond number variation during simulation; (J) detailed binding mode and intermolecular interaction of EGFR-carbamazepine complex.

**Supplementary Figure 3. Molecular dynamics simulation analysis of PB-CASP3 complex.Molecular dynamics simulation analysis of EGFR-carbamazepine complex Subgraph description：(A) RMSD of complex, protein and ligand; (B) Rg of protein; (C) RMSF of each residue; (D) average distance between pocket-ligand and protein-ligand; (E) buried SASA; (F) protein conformational clustering; (G) binding energy decomposed into electrostatic (ELE) and van der Waals (VDW) components; (H) residue-wise binding energy contribution; (I) hydrogen bond number variation during simulation; (J) detailed binding mode and intermolecular interaction of PB-** **CASP3 complex.**

**Supplementary Figure 4.** Molecular dynamics simulation analysis of PHT- PPARG complex.(A) RMSD of complex, protein and ligand; (B) Rg of protein; (C) RMSF of each residue; (D) average distance between pocket-ligand and protein-ligand; (E) buried SASA; (F) protein conformational clustering; (G) binding energy decomposed into electrostatic (ELE) and van der Waals (VDW) components; (H) residue-wise binding energy contribution; (I) hydrogen bond number variation during simulation; (J) detailed binding mode and intermolecular interaction of PHT- PPARG complex.

**Supplementary Figure 5. Molecular dynamics simulation analysis of VPA -** **PTGS2 complex.** (A) RMSD of complex, protein and ligand; (B) Rg of protein; (C) RMSF of each residue; (D) average distance between pocket-ligand and protein-ligand; (E) buried SASA; (F) protein conformational clustering; (G) binding energy decomposed into electrostatic (ELE) and van der Waals (VDW) components; (H) residue-wise binding energy contribution; (I) hydrogen bond number variation during simulation; (J) detailed binding mode and intermolecular interaction of VPA - PTGS2 complex.

**Supplementary Figure 6. Cell viability measurement via CCK-8 assay after exposure to various antiepileptic drugs. Scatter dot plots illustrate corrected OD_450_ values reflecting cell viability following 24 h or 48 h drug treatment. (A,B) Cells treated with carbamazepine (20 μM and 40 μM) for 24 h and 48 h, respectively. (C,D) Cells treated with phenytoin sodium (10 μM and 20 μM) for 24 h and 48 h, respectively. (E,F) Cells treated with phenobarbital (20 μM and 40 μM) for 24 h and 48 h, respectively. (G,H) Cells treated with sodium valproate (500 μM and 800 μM) for 24 h and 48 h, respectively. Each dot denotes an independent biological replicate. ns, not significant.**

****Supplementary Files S1**：Original molecular docking source files for the four drug–target complexes.**

****Supplementary Files S2**：Full Uncropped Western Blot Independent Replicates.**

**Supplementary Table S1：Target Information Screened from Multiple Databases.**

**Supplementary Table S2：Detailed information of shared candidate targets.**

**Supplementary Table S3：The complete results of functional enrichment analysis.**

**Supplementary Table S4：Protein structure information for molecular docking.**

**Supplementary Table S5：Molecular docking parameters and screening results for antiepileptic drug–target protein complexes.**

**Supplementary Table S6：System dimensions and composition of molecular dynamics simulation boxes.**

**Supplementary Table S7：Information regarding cells and culture conditions.**

**Supplementary Table S8：Detailed antibody information for protein detection.**

**Supplementary Table S9：Summary of MD Input Parameters and Output Data for Four Drug-Target Complexes.**

**Supplementary Table S10：Results of CCK-8 assay for cytotoxicity of AEDs.**

**Supplementary Table S11：Cell proliferation inhibition statistics of four antiepileptic drugs at different concentrations in colony formation assay.**

**Supplementary Table S12：Protein band gray intensity.** **Raw gray intensity values for protein bands detected by western blot. Band gray intensities were quantified for subsequent relative expression calculation.**
